# 

**Published:** 1911-02-04

**Authors:** 


### THE HOSPITAL

### THE MODERN MEDICAL AND INSTITUTIONAL JOURNAL.

Telegraphic Address: Ospedale, London.

Telephone Number: 2734 Gerrard.

NEW SERIES. No. 209, Vol. VIII.

No. 1281, Vol. XLIX.

SATURDAY, FEB. 4TH, 1911.

PRICE THREEPENCE

